# Diet and Chronic Diseases: Is There a Mediating Effect of Inflammation?

**DOI:** 10.3390/nu11071639

**Published:** 2019-07-18

**Authors:** Nitin Shivappa

**Affiliations:** 1 South Carolina Statewide Cancer Prevention and Control Program, University of South Carolina, Columbia, SC 29208, USA; shivappa@email.sc.edu; 2 Department of Epidemiology and Biostatistics, University of South Carolina, Columbia, SC 29208, USA; 3 Connecting Health Innovations LLC, Columbia, SC 29201, USA

**Keywords:** dietary inflammatory index, inflammation, cardiovascular disease, non-communicable diseases

Chronic non-communicable diseases like cardiovascular disease (CVD) and diabetes represent the majority of the current burden of disease worldwide, with higher rates and impacts in developed countries but also with alarming trends in developing countries [1,2]. Among the major risk factors, dietary components have been recently shown to play a crucial role, with a double burden of malnutrition affecting both developed and developing countries [1,3]; the risks associated with malnutrition, intended not only as over-nutrition but also as poor diet quality, have been reported to significantly impact not only cardio-metabolic disorders (far more studied over the last decades), but also cancer and certain aspects related to mental health [4,5]. All these groups of diseases, in spite of the different organs and systems involved, disability and prognosis impacted, have been hypothesized and partially demonstrated to have a common risk factor, or starting point, in general low-grade, clinically silent inflammation [6]. Such inflammatory state is supposed to be assessed through testing for biomarkers, such as pro-inflammatory cytokines, and may be caused by a number of conditions, including, but not limited to, obesity (pro-inflammatory cytokines are produced by an excess of adipose tissue), active smoking, and exercising [7,8,9]. These observations provided the rationale for the hypothesis that diet may modify the risk of major chronic non-communicable diseases by affecting the inflammatory status [10].

Dietary inflammatory index (DII^®^) is a literature-derived dietary tool developed by Shivappa and his colleagues to determine the inflammatory potential of an individual’s entire diet as opposed to individual nutrients or food components. In developing the DII, around 2000 peer-reviewed scholarly articles that examined the association between 45 dietary components and inflammation were reviewed and scored [11,12]. Within this list there are several macro- and micronutrients; food items such as garlic, ginger, and onions; and important bioactive polyphenols such as flavonoids. DII has previously been validated with several inflammatory markers [13,14,15,16] and has been shown to be associated with several health outcomes [17,18,19,20,21,22].

Several summary articles have also been produced to quantitatively synthesize the evidence on the association between the DII and the risk of various conditions [12]. Among the most recent articles, we conducted a systematic review and meta-analysis on the association between the DII and cardiovascular disease risk, in which we found an 8% increased risk of CVD incidence and mortality for each 1-point increase of the score [23]. The results of the meta-analysis support the importance of adopting a healthier anti-inflammatory diet that is rich in polyphenols like flavonoids in preventing CVD incidence and related mortality. In conclusion, a pro-inflammatory diet is associated with increased risk of CVD and CVD mortality. Although the exact mechanism behind this association is not known, a few theories have been proposed; one of them is through the effect of pro-inflammatory diet on increasing levels of inflammatory cytokines which, in turn, results in the migration and accumulation of inflammatory cells into vascular tissue [24], which can eventually result in damage to vascular tissue.

Other studies have been conducted lately, including studies summarizing the evidence of the inflammatory potential of diet increasing the risk of depression [25], certain cancers, such as gynecological, urological, and colorectal cancers [26,27,28], and mortality [29,30]. Future studies further elucidating and describing the mechanistic processes involved with a pro-inflammatory diet and the development of chronic non-communicable diseases would provide definitive evidence for the usability of the DII tool and its application in clinical setting for testing current diets and, eventually, for promoting improvements to diet quality.
